# The Longitudinal Relationship between Jaw Catching/Locking and Pain

**DOI:** 10.1177/00220345221138532

**Published:** 2022-12-01

**Authors:** A. Ilgunas, B. Häggman-Henrikson, C.M. Visscher, F. Lobbezoo, J. Durham, P. Liv, A. Lövgren

**Affiliations:** 1 Department of Odontology/Clinical Oral Physiology, Faculty of Medicine, University of Umeå, Umeå, Sweden; 2 Department of Orofacial Pain and Jaw function, Faculty of Odontology, Malmö University, Malmö, Sweden; 3 Department of Orofacial Pain and Dysfunction, Academic Centre for Dentistry Amsterdam (ACTA), University of Amsterdam and Vrije Universiteit Amsterdam, Amsterdam, The Netherlands; 4 School of Dental Sciences, Newcastle University, Newcastle, UK; 5 Newcastle Hospitals’ NHS Foundation Trust, Newcastle, UK; 6 Section of Sustainable Health, Department of Public Health and Clinical Medicine, University of Umeå, Umeå, Sweden

**Keywords:** cohort studies, dentistry, epidemiology, facial pain, population health, temporomandibular joint disorders

## Abstract

Orofacial pain and joint-related dysfunction can negatively affect daily jaw function. A common cause for limitations in jaw movements is joint-related dysfunction such as various forms of catching and locking. However, knowledge is limited regarding the development and natural course of joint-related jaw dysfunction and its relationship to the onset and course of orofacial pain. Therefore, the aim was to evaluate the incidence, prevalence, and gender differences in jaw catching/locking over time and in relation to orofacial pain in the general population. Data from 3 validated screening questions on orofacial pain and jaw catching/locking were collected from all routine dental checkups in the Public Dental Health Services in Västerbotten, Sweden, from 2010 to 2017. Logistic generalized estimating equation was used to account for repeated observations and Poisson regression for incidence analysis. In total, 180,308 individuals (aged 5–104 y) were screened in 525,707 dental checkups. In 2010, based on 37,647 individuals, the prevalence of self-reported catching/locking was higher in women than in men (3.2% vs. 1.5%; odds ratio, 2.11; 95% confidence interval [CI], 1.83–2.43), and this relationship and magnitude remained similar throughout the study period. The annual incidence rate was 1.1% in women and 0.5% in men. Women were at a higher risk than men for reporting both first onset (incidence rate ratio [IRR], 2.29; 95% CI, 2.11–2.49) and persistent (IRR, 2.31; 95% CI, 2.04–2.63) catching/locking. For the onset subcohort (*n* = 135,801), an independent onset of orofacial pain or jaw catching/locking exclusively was reported by 84.1%, whereas a concurrent onset was reported by 13.4%. Our findings of higher incidence, prevalence, and persistence in women than in men indicate that the gender differences seen for orofacial pain are evident also for jaw catching/locking. The findings also suggest independent onset of self-reported catching/locking and orofacial pain, which reinforces the pathophysiological differences between these conditions.

## Introduction

Temporomandibular disorder (TMD) is the term used to embrace orofacial pain and jaw dysfunction involving the masticatory muscles, the temporomandibular joint (TMJ), and associated structures (Dworkin and LeResche 1992; LeResche 1997). Due to its chronic nature (Maixner et al. 2011) and frequent comorbidities (Ohrbach et al. 2011; Sanders et al. 2013), TMD symptoms negatively affect oral health–related quality of life (Suvinen et al. 2005; Dahlström and Carlsson 2010). TMD affects 10% to 15% of the general adult population, with the highest prevalence among individuals 20 to 50 y of age. Women have twice the prevalence of TMD as men (Isong et al. 2008; Manfredini et al. 2011; Lövgren, Häggman-Henrikson, et al. 2016) and, as for most other pain conditions, are also at higher risk to develop chronic orofacial pain (Macfarlane et al. 2001). In a longitudinal study, we recently reported gender differences in the development and chronification of orofacial pain (Häggman-Henrikson et al. 2020), but the joint-related conditions and their relationship to pain were not investigated.

Orofacial pain, muscle-related problems, and TMJ-related conditions can impair jaw activities. Thus, individuals with TMD report a variety of symptoms, including pain, fatigue, and jaw clicking and locking, that often negatively affect daily jaw function. Joint-related disorders such as degenerative joint disease and disc displacements—with or without reduction—may manifest as jaw catching and locking in addition to the more common clicking sounds. Joint sounds, however, are prevalent also in nonpatient populations (Valesan et al. 2021) and are therefore considered predominately a physiological variation, whereas catching and locking can impair jaw function, including communication and mastication (Ohrbach et al. 2008). Although TMJ catching and locking are less common (Manfredini et al. 2011) and less explored than orofacial pain, the impact on the individual is often substantial and is therefore important to evaluate.

Studies on the relationship between joint-related jaw dysfunction and orofacial pain report conflicting results-for example, frequent presence of pain (Emshoff and Rudisch 2003) versus absence of pain (Chantaracherd et al. 2015) in joint-related conditions. Understanding how joint-related jaw dysfunction and pain develop in relation to each other over time is fundamental in evaluating both risk factors and prognosis, as well as in the planning of treatment. However, knowledge on this relationship, especially in a longitudinal perspective, is currently limited.

Longitudinal studies from the general population may provide crucial information about the development of joint-related jaw dysfunction over time and its relationship to the onset of orofacial pain. Therefore, the aim was to evaluate the incidence, prevalence, and gender differences in jaw catching/locking over time and in relation to orofacial pain in the general population. We hypothesized that jaw catching/locking is more frequent in women than in men. We also hypothesized that the onset of jaw catching/locking is predominantly concurrent with the onset of orofacial pain.

## Materials and Methods

### Study Setting

The present study was conducted from May 2010 to December 2017 at the Public Dental Health Services (PDHS) in the Region of Västerbotten, Sweden. Dental care in Sweden is provided both by the PDHS and by private practitioners, and it is government subsidized, regardless of whether the patient visits a PDHS or a private dental practitioner. Most of the Swedish population (80%) undergo dental examinations regularly. The minority of the population who do not have regular routine dental examinations often have lower socioeconomic status and general health issues (National Board of Health and Welfare 2018). Routine dental examinations are performed by dentists or by dental hygienists, and in the Region of Västerbotten, a digital health declaration is completed during these appointments. The health declaration includes 3 validated mandatory screening questions for frequent TMD symptoms, the 3Q/TMD (Lövgren, Visscher, et al. 2016). The questions are formulated as follows:

Q1: Do you have pain in your temple, face, jaw, or jaw joint once a week or more?
Q2: Do you have pain once a week or more when you open your mouth or chew?
Q3: Does your jaw lock or become stuck once a week or more?

The first 2 questions identify orofacial pain, and the third question identifies jaw catching/locking. The answers, either yes or no, are provided either by the patients themselves or by parents or guardians if the patient has difficulties understanding or answering the questions. All data are stored in a local database in the Region of Västerbotten. Occasionally, the health declaration is not filled in and therefore the answers to the 3Q/TMD are missing. In the sample for the present study, 13% of the data were missing. Individuals with affirmative answers to Q3 were classified as cases for catching/locking, and individuals with affirmative answers to Q1 and/or Q2 were classified as cases for orofacial pain.

### Study Population

**Figure 1.**
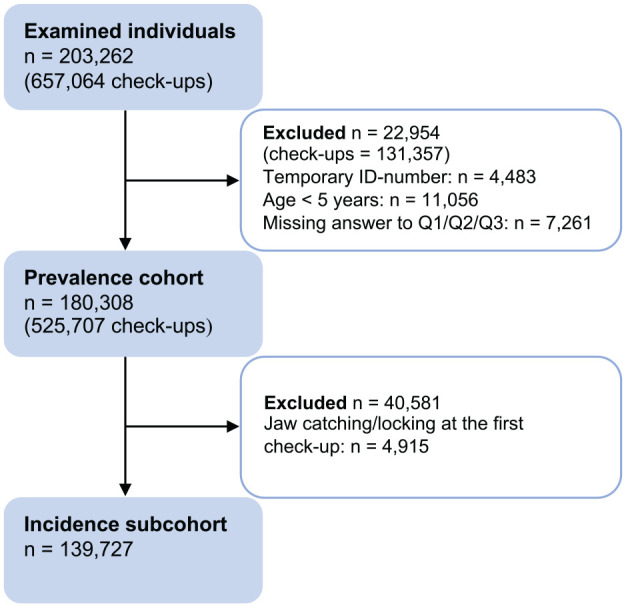
The flowchart of the study population. Excluded individuals may be present in more than 1 of the subgroups.

All individuals aged 5 y or older, who underwent a routine dental examination at PDHS in the Region of Västerbotten and had a completed digital health declaration that included 3Q/TMD, were enrolled in the study (Fig. 1). In total, 180,308 individuals (equal gender distribution) were examined in 525,707 dental examinations (median age at examination: 29.0 y; interquartile range [IQR], 16–29 y), with a median number of 3 examinations per individual over the study period (Table 1). Individuals with a temporary personal identity number were excluded as this made longitudinal follow-ups impossible.

**Table 1.** Demographical Characteristics of the Total Study Sample Examined from May 2010 to December 2017 in the Region of Västerbotten, Sweden, and the Number of Examinations for Each Individual over the 8-Year Period for the Whole Sample Group (*n* = 180,308), for the Subsample Included in the Incidence Analysis (*n* = 139,727), and among the Individuals with the Persistent Symptoms (*n* = 104,644).

Variable	Sample, *n*	Age, y		Examinations for Each Individual, *n* (%)							
		Mean (SD)	Median (IQR)	1	2	3	4	5	6	7	8
Total	180,308	34.3 (22.7)	29.0 (16.0, 29.0)	38,026 (21.1)	35,794 (19.9)	42,724 (23.7)	38,518 (21.4)	18,921 (10.5)	5,165 (2.9)	1,041 (0.6)	119 (0.1)
Women	90,166	34.9 (23.0)	30.0 (15.0, 30.0)	19,071 (21.2)	18,015 (20.0)	21,392 (23.7)	19,196 (21.3)	9,347 (10.4)	2,539 (2.8)	540 (0.6)	66 (0.1)
Men	90,142	33.7 (22.4)	29.0 (15.0, 29.0)	18,955 (21.0)	17,779 (19.7)	21,332 (23.7)	19,322 (21.4)	9,574 (10.6)	2,626 (2.9)	501 (0.6)	53 (0.1)
Incidence cohort											
Total	139,727	33.1 (22.5)	28.0 (14.0, 28.0)	NA	35,083 (25.1)	41,945 (30.0)	37,825 (27.1)	18,641 (13.3)	5,095 (3.6)	1,021 (0.7)	117 (0.1)
Women	69,360	33.7 (22.8)	29.0 (14.0, 28.0)	NA	17,545 (25.3)	20,854 (30.1)	18,726 (27.0)	9,155 (13.2)	2,490 (3.6)	526 (0.8)	64 (0.1)
Men	70,367	32.6 (22.2)	27.0 (14.0, 27.0)	NA	17,538 (24.9)	21,091 (30.0)	19,099 (27.1)	9,486 (13.5)	2,605 (3.7)	495 (0.7)	53 (0.1)
Persistent cohort											
Total	104,644	32.5 (22.4)	27.0 (13.0, 27.0)	NA	NA	41,945 (40.1)	37,825 (36.1)	18,641 (17.8)	5,095 (4.9)	1,021 (1.0)	117 (0.1)
Women	51,815	33.0 (22.7)	27.0 (13.0, 27.0)	NA	NA	20,854 (40.2)	18,726 (36.1)	9,155 (17.7)	2,490 (4.8)	526 (1.0)	64 (0.1)
Men	52,829	32.0 (22.0)	26.0 (13.0, 26.0)	NA	NA	21,091 (39.9)	19,099 (36.2)	9,486 (18.0)	2,605 (4.9)	495 (0.9)	53 (0.1)

IQR, interquartile range (first and third quartiles); NA, not applicable; SD, standard deviation.

### Incidence Subcohort

Longitudinal data on 139,727 individuals with no self-reported catching/locking at the first dental examination were available for the incidence analysis (Table 1). Annual follow-up was rare; therefore, only years when an individual had an examination contributed to the calculation of total person-years. At the first affirmative answer to Q3, an individual was categorized as a case with first onset of jaw catching/locking. An affirmative answer to Q3 on at least 2 consecutive examinations categorized an individual as a case with persistent jaw catching/locking.

### Onset Subcohort

Individuals with no self-reported jaw catching/locking or orofacial pain at their first dental examination and at least 2 further dental examinations during the study period were potential cases and included in the descriptive onset analysis (*n* = 135,801). At the first examination with reported symptoms, individuals were recategorized as cases with onset of jaw catching/locking, orofacial pain, or both. These cases were followed until their last dental examination, and the following symptoms were recorded: independent and exclusive, without the other symptom, onset of catching/locking or orofacial pain, concurrent onset of catching/locking and orofacial pain, a first onset of catching/locking and a later onset of orofacial pain, or a first onset of orofacial pain and a later onset of catching/locking.

### Statistical Methods

Data analysis was performed using the statistical software R v.3.5.3 (R Core Team 2019). Descriptive statistics were used to characterize the study population and to estimate the relationship between the onset of jaw catching/locking and/or orofacial pain. The cohort and subcohorts were characterized by the number of available examinations per individual and the median and interquartile range of age at first visit. The 12-mo prevalence of jaw catching/locking was calculated annually, separately for women and men. Generalized estimating equation models with logit link function were used to analyze the prevalence of jaw catching/locking, with age as an independent variable. Age was modeled using natural cubic splines with 5 knots at the 16.7th, 33.3rd, 50th, 66.7th, and 83.3rd percentiles of the study population’s age distribution. Splines is a method for fitting smooth regression lines and allows the model to account for a nonlinear relationship between age and prevalence of jaw catching/locking, as this was previously shown to exist by Lövgren and coworkers (Lövgren, Häggman-Henrikson, et al. 2016). The models were fitted stratified for gender and fitted on the full cohort, including gender as a factor.

Incidence rates for first onset and persistent jaw catching/locking were calculated as the ratio between the number of new cases during the follow-up period and the total number of person-years at risk. Incidence rate ratios (IRRs) between women and men were calculated using Poisson regression and adjusted for age at the first dental examination employing natural cubic splines with 3 knots at the 10th, 50th, and 90th percentiles. Odds ratios (ORs) were presented with 95% confidence intervals (CIs). The significance level was set at 0.05.

The study was approved by the Ethical Board at Umeå University (reference 2012-331-31M, 2018-181-32M, and 2018/393-3) and conformed to the Strengthening the Reporting of Observational Studies in Epidemiology (STROBE) guidelines (von Elm et al. 2014) (Appendix Table 1).

## Results

### Prevalence of Jaw Catching/Locking

**Figure 2.**
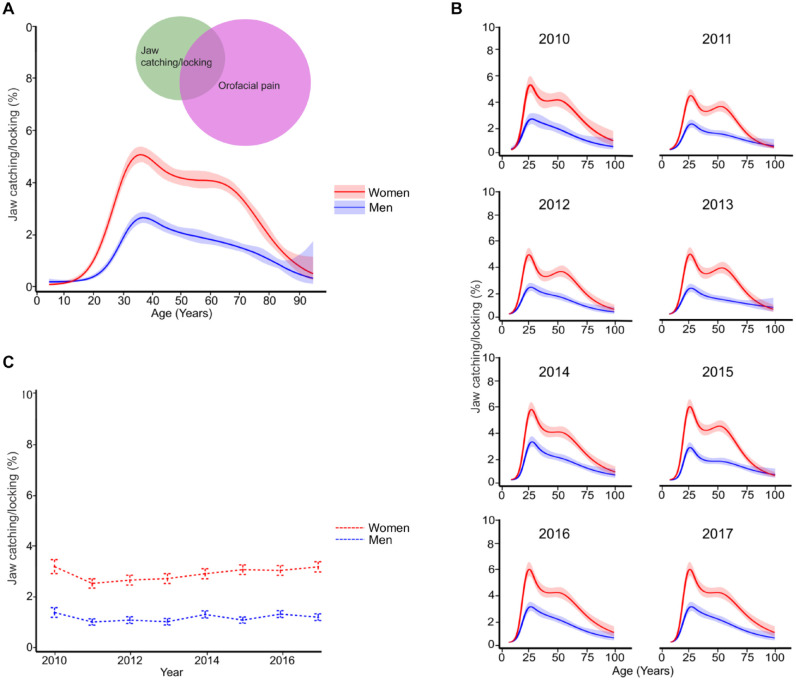
The estimated prevalence of jaw catching/locking (95% confidence interval) in women and men. (**A**) As a function of age, 2010 to 2017 together with a Venn diagram of the overlap with orofacial pain. (**B**) As a function of age for individual years. (**C**) For individual years for the whole study period.

In 2010, the prevalence of self-reported jaw catching/locking was significantly higher in women than in men (3.2% vs. 1.5%; OR, 2.11; 95% CI, 1.83–2.43) (Table 2). The prevalence pattern for women showed 2 peaks over the life span—the first in the late 20s and the second in the 60s (Fig. 2A, B). For both women and men, the highest prevalence of jaw catching/locking was in the 20- to 30-y age group with a consistent pattern over the 8-y study period (Fig. 2B). There was no significant change in the prevalence of jaw catching/locking from 2010 to 2017 (Table 2; Fig. 2C).

**Table 2.** The 1-Year Period Prevalence of Jaw Catching/Locking with 95% Confidence Interval for the Full Study Period, Stratified by Gender.

Year	Coverage (%)	Women			Men		
		*n*	Cases, *n*	Prevalence (95% CI)	*n*	Cases, *n*	Prevalence (95% CI)
2010	51.7^a^	18,659	596	3.2 (2.8–3.5)	18,988	292	1.5 (1.1–1.9)
2011	88.8	34,058	881	2.6 (2.3–2.8)	34,505	412	1.2 (0.9–1.5)
2012	89.3	32,183	871	2.7 (2.4–2.9)	32,568	411	1.3 (0.9–1.6)
2013	90.3	33,050	913	2.8 (2.5–3.1)	33,131	399	1.2 (1.0–1.5)
2014	92.6	33,504	984	2.9 (2.6–3.2)	33,545	492	1.5 (1.2–1.8)
2015	93.9	38,426	1,185	3.1 (2.8–3.4)	38,400	485	1.3 (1.0–1.5)
2016	95.0	37,307	1,140	3.1 (2.8–3.4)	37,294	552	1.5 (1.2–1.8)
2017	95.0	35,159	1,120	3.2 (2.9–3.5)	34,930	478	1.4 (1.1–1.7)

The proportion of screened individuals at regular dental checkups for each year is given as a percentage of data coverage. The individuals who screened positive to jaw catching/locking are identified as cases.

CI, confidence interval.

a The 3Q/TMD was first introduced to the Public Dental Health Services in 2010. The implementation process was stepwise; therefore, the coverage rate is lower when compared to the subsequent years.

### Incidence of Jaw Catching/Locking

The annual incidence rate of reported jaw catching/locking was 1.1% in women and 0.5% in men. For persistent catching/locking, the annual incidence rate was 0.5% in women and 0.2% in men. Women were at a higher risk than men for reporting first onset catching/locking (IRR, 2.29; 95% CI, 2.11–2.49) and for reporting catching/locking in consecutive dental examinations (IRR, 2.32; 95% CI, 2.04–2.63).

### Overlap of Jaw Catching/Locking and Orofacial Pain

In total, 29,261 (5.1%) dental examinations with reported jaw catching/locking or orofacial pain from the study cohort were available for the descriptive relationship analysis. Orofacial pain in the absence of catching/locking was reported by 61.7% (62.8% women, 58.9% men) of dental examinations, while catching/locking in the absence of pain was reported by 22.0% (17.8% women, 28.4% men). An overlap (i.e., concurrent catching/locking and orofacial pain) was reported by 16.3% (17.8% women, 12.7% men) of dental examinations (Fig. 2A).

### Onset of Jaw Catching/Locking and Orofacial Pain

**Figure 3.**
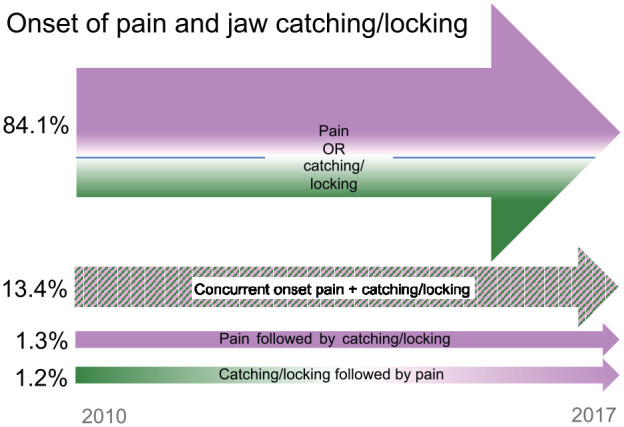
The onset of orofacial pain (magenta) and jaw catching/locking (green) based on 6,594 individuals during 2010 to 2017.

In total, data from 6,594 individuals (68.9% women, 31.1% men) were available for descriptive onset analysis. The onset of orofacial pain as an exclusive symptom during the study period was reported by 64.9% (65.5% women, 63.7% men). The onset of jaw catching/locking as an exclusive symptom during the study period was reported by 19.2% (17.3% women, 23.2% men). The onset of orofacial pain followed by a later onset of catching/locking was reported by 1.3% (1.2% women, 1.3% men), while the onset of catching/locking followed by a later onset of orofacial pain was reported by 1.2% (1.5% women, 0.7% men). A concurrent onset of catching/locking and orofacial pain was reported by 13.4% (14.5% women, 11.1% men) (Fig. 3).

## Discussion

This study showed that the onset of jaw catching/locking independent of the onset of orofacial pain is the predominant occurrence in the general population. The prevalence of reported jaw catching/locking was higher in women when compared to men, and women were also at a higher risk for reporting both first onset and persistent catching/locking. Furthermore, men reported jaw catching/locking as an independent symptom, hence without pain, more frequently than women, thus indicating an overall higher burden of orofacial pain in women. Our hypothesis on jaw catching/locking being more frequent in women than men was therefore accepted. However, our hypothesis regarding a concurrent onset of catching/locking and orofacial pain was rejected.

The large-scale data from the general population over an 8-y period presented in this study are unique for research on orofacial pain and joint-related jaw dysfunction, albeit with limitations related to the study design that may negatively influence generalizability of the results. Nevertheless, and even though reports on prevalence of oral health, including orofacial pain, differ largely worldwide, a recent report on pain prevalence in 52 countries places Sweden at a global average (Zimmer et al. 2022). Given that country-level differences in prevalence are related to cultural, social, and economic factors (Zimmer et al. 2022), we regard our results valid for similar settings as ours.

The necessity of brief, yet reliable and valid, screening and diagnostic instruments for orofacial pain and TMD has been highlighted, and the use of such instruments has been encouraged (Lobbezoo, Aarab, Kapos, Dayo, Huang, et al. 2022; Lobbezoo, Aarab, Kapos, Dayo, Koutris, et al. 2022). By using the 3Q/TMD screening instrument, data from nearly 200,000 individuals were available for analysis. In previous studies, we observed considerable overlap between Q1 and Q2 (Häggman-Henrikson et al. 2020), as well as the highest sensitivity for a TMD pain diagnosis when either of these questions was answered affirmatively (Lövgren, Visscher, et al. 2016). Therefore, in the present study, we used Q1 and Q2 in combination as representative for orofacial pain and Q3 (catching or locking) as representative for self-reported joint-related jaw dysfunction. It should be noted, however, that joint-related jaw dysfunction includes not only catching/locking but also other symptoms such as clicking (Manfredini et al. 2011). From a clinical perspective, joint-related conditions may be challenging to diagnose, and even a clinical evaluation has limitations in diagnostic accuracy (Schiffman et al. 2014). Therefore, magnetic resonance imaging and computed tomography are regarded as the gold-standard method to reliably evaluate intra-articular conditions (Schiffman et al. 2010). However, a clinical examination, or indeed imaging, in a large sample like ours would clearly be too time-consuming and expensive to be feasible. Thus, the use of the validated screening tool 3Q/TMD in the present study presented the best available method for cost-effective research of orofacial pain and jaw catching/locking in a large population sample.

Age-related difficulties can occur in understanding and answering questions for the youngest or oldest participants. However, as age-related barriers are more common for questions with higher response option difficulty than in those with dichotomous answers (Knauper et al. 2016), this risk is reduced in the present study by the use of dichotomous screening questions. Furthermore, the youngest and oldest patients were usually accompanied by the parents or guardians who could assist with the interpretation and answering of the questions.

The county of Västerbotten consists of nearly 270,000 inhabitants, of whom approximately 180,000 (70%) have regular routine dental examinations, and more than half (54%) of these examinations are performed by the PDHS (National Board of Health and Welfare 2018). In the present setting, we had a coverage of more than half of the population in Västerbotten, which is well in line with acceptable coverage for surveys in general of 50% to 60% (Nulty 2008). The frequency of the routine dental examinations in Sweden varies from approximately once per year to once every 2 or 3 y. This depends not only on a patient’s own initiative but also on the dental health of the patient as determined by the dentist. In the present study, the median number per individual was 3 dental examinations over the 8-y period. Extrapolation of the findings outside the study’s follow-up period should be done with caution, but collectively we believe our results are generalizable for comparable settings.

The relationship between the onset of joint-related jaw dysfunction and pain has not been fully elucidated previously, and our study is the first to explore this relationship in a large sample over time. We found that the respective onsets of self-reported orofacial pain and jaw catching/locking were most frequently independent and exclusive, which reinforces differences in the pathophysiology for these conditions. The theories of heightened symptom awareness and hypervigilance consider that mechanical factors, including jaw locking, are reported more commonly by both individuals with orofacial pain and those with widespread pain (Macfarlane et al. 2002). In addition, higher levels of kinesiophobia and catastrophizing are reported by individuals with both painful and nonpainful TMDs (Häggman-Henrikson et al. 2022). Such findings suggest the overlap between orofacial pain and jaw dysfunction as more of a symptom due to the increased awareness than as a shared pathophysiological path.

In contrast to our previous findings of an increase of orofacial pain (Häggman-Henrikson et al. 2020), we found no increase over time in the prevalence of self-reported jaw catching/locking. This finding is in agreement with previous longitudinal studies that employed diagnostic imaging for the evaluation of intra-articular changes. Thus, approximately 70% of baseline intra-articular conditions such as disc displacement and degenerative joint disease remained stable at an 8-y follow-up (Schiffman et al. 2017), and neither increase in prevalence nor significant progression of disk displacement was observed in a 15-y follow-up (Salé et al. 2013).

In our study, the highest prevalence of jaw catching/locking over the life span was consistently in the 20- to 30-y age group, and the prevalence formed a consistent twin peak pattern in women over the study period. Several studies have showed that the overall TMD prevalence is highest in the 20- to 40-y age group and is higher in women (Progiante et al. 2015; Lövgren, Häggman-Henrikson, et al. 2016). The observed twin peaks in women are also in line with a previous study on the prevalence of TMD over the life span based on clinical examinations (Guarda-Nardini et al. 2012). In their study, 1 peak was found at the age of 38 y for patients with pain and/or degenerative disc disorders but without joint crepitus and a second peak at the age of 52 y for patients with crepitus (Guarda-Nardini et al. 2012). This is corroborated by reports that disc displacement is frequent in the younger ages, whereas degenerative joint disease is more prevalent among older individuals (Rutkiewicz et al. 2006; Yadav et al. 2018). It is therefore reasonable to assume that the self-reported jaw catching/locking in our study mainly represents disc displacement in the first age peak and degenerative joint disease in the second age peak. Furthermore, the results suggest that on a population level, there seems to be a stability in jaw catching/locking over time regarding both overall prevalence and the age groups with the highest prevalence.

Our study also demonstrated a female preponderance for self-reported incidence and prevalence of jaw catching/locking. Such gender differences could to some extent be related to comorbidities (Sanders et al. 2013), with catching/locking being part of a general health impairment in women. For example, other joint-related conditions such as joint hypermobility are more prevalent in women than men (Remvig et al. 2007) and were shown to be related to nonpainful TMD (i.e., joint-related disorders) (Hirsch et al. 2008). The biopsychosocial model has been used to explain the etiology and related gender differences in TMD (Suvinen et al. 2005). As one of the biological components, hormonal fluctuations have been discussed where estrogen levels were suggested to affect TMJ degeneration (Yadav et al. 2018). However, gender differences in health may be more closely related to socioeconomic and behavioral factors rather than anatomical or genomic differences, as emphasized by emerging research on sex-stratified medicine (Westergaard et al. 2019). Our finding of no difference between women and men in the onset of catching/locking could be related to similarities in anatomical features of the jaw joint, whereas the gender differences for concurrent pain with catching/locking may be related to pain-related behavioral factors such as higher levels of health awareness (Ek 2015) and health care seeking (Thompson et al. 2016) in women as compared to men. In this regard, future research on equity in the management of patients with TMD through the lens of gender and socioeconomic factors would be valuable.

## Conclusions

In conclusion, our findings of higher incidence, prevalence, and persistence of jaw catching/locking in women than in men indicate that the gender differences seen for orofacial pain are also evident for self-reported catching/locking. The findings also suggest independent onset of jaw catching/locking and pain, which reinforces that the pathophysiology differs between these conditions.

## Author Contributions

A. Ilgunas, B. Häggman-Henrikson, P. Liv, A. Lövgren, contributed to conception and design, data analysis and interpretation, drafted and critically revised the manuscript; C.M. Visscher, contributed to conception and design, data analysis and interpretation, critically revised the manuscript; F. Lobbezoo, J. Durham, contributed to conception and design, data interpretation, critically revised the manuscript. All authors gave final approval and agree to be accountable for all aspects of the work.

## Supplemental Material

sj-docx-1-jdr-10.1177_00220345221138532 – Supplemental material for The Longitudinal Relationship between Jaw Catching/Locking and Pain Click here for additional data file. Supplemental material, sj-docx-1-jdr-10.1177_00220345221138532 for The Longitudinal Relationship between Jaw Catching/Locking and Pain by A. Ilgunas, B. Häggman-Henrikson, C.M. Visscher, F. Lobbezoo, J. Durham, P. Liv and A. Lövgren in Journal of Dental Research
