# Supplementary material for: The Longitudinal Relationship between Jaw Catching/Locking and Pain
Source: J Dent Res. 2022 Dec 1;102(4):383–90. doi: 10.1177/00220345221138532 (PMC10031631; doi:10.1177/00220345221138532)
Supplement: sj-docx-1-jdr-10.1177_00220345221138532 – Supplemental material for The Longitudinal Relationship between Jaw Catching/Locking and Pain [file sj-docx-1-jdr-10.1177_00220345221138532.docx]

Appendix

The Longitudinal Relationship between Jaw Catching/Locking and Pain

*Aurelia Ilgunas, Birgitta Häggman-Henrikson, Corine M Visscher, Frank Lobbezoo, Justin Durham, Per Liv, Anna Lövgren*

Appendix Table 1

|  | Item No. | Recommendation | Page  No. | Relevant text from manuscript |
| --- | --- | --- | --- | --- |
| **Title and abstract** | 1 | (*a*) Indicate the study’s design with a commonly used term in the title or the abstract | Title and abstract | Longitudinal study |
|  |  | (*b*) Provide in the abstract an informative and balanced summary of what was done and what was found | Abstract | What was done: Data from three validated screening questions on orofacial pain and jaw catching/locking were collected from all routine dental check-ups in the Public Dental Health Services in Västerbotten, Sweden, from 2010 to 2017  What was found: higher incidence, prevalence, and persistence of jaw catching/locking in women than in men. The exclusive onsets of self-reported jaw catching/locking or orofacial pain. |
| Introduction | | | |  |
| Background/rationale | 2 | Explain the scientific background and rationale for the investigation being reported | 3-4 | Although catching and locking are less common and less explored than pain, the impact on the individual is often substantial and is therefore important to evaluate. (…) the joint-related conditions and their relationship to pain were not investigated. (…) Studies on the relationship between joint-related jaw dysfunction and orofacial pain report conflicting results (…) Understanding how joint-related jaw dysfunction and pain develop in relation to each other over time is fundamental in evaluating both risk and prognosis, as well as in the planning of treatment. |
| Objectives | 3 | State specific objectives, including any prespecified hypotheses | 4 | The aim was to evaluate the incidence, prevalence, and gender differences in jaw catching/locking over time and in relation to orofacial pain in the general population. We hypothesized that jaw catching/locking is more frequent in women than in men. We also hypothesized that the onset of jaw catching/locking is predominantly concurrent with the onset of orofacial pain. |
| Methods | | | |  |
| Study design | 4 | Present key elements of study design early in the paper | 4 | Population-based sample. Self-reported symptoms. Longitudinal data. |
| Setting | 5 | Describe the setting, locations, and relevant dates, including periods of recruitment, exposure, follow-up, and data collection | 4-5 | 2010-2017, the Region of Västerbotten. Public dental health services. Self-reported data from the screening instrument 3Q/TMD. |
| Participants | 6 | (*a*) *Cohort study*—Give the eligibility criteria, and the sources and methods of selection of participants. Describe methods of follow-up  *Case-control study*—Give the eligibility criteria, and the sources and methods of case ascertainment and control selection. Give the rationale for the choice of cases and controls  *Cross-sectional study*—Give the eligibility criteria, and the sources and methods of selection of participants | 5, Figure 1 | All individuals aged five or older, who underwent a routine dental examination at PDHS in the Region of Västerbotten and had a completed digital health declaration that included 3Q/TMD, were enrolled in the study. The youngest and the oldest individuals usually had support and help to understand the questions. |
|  |  | (*b*) *Cohort study*—For matched studies, give matching criteria and number of exposed and unexposed  *Case-control study*—For matched studies, give matching criteria and the number of controls per case | N/A |  |
| Variables | 7 | Clearly define all outcomes, exposures, predictors, potential confounders, and effect modifiers. Give diagnostic criteria, if applicable | 5-6 | Gender, age, calendar years. |
| Data sources/ measurement | 8* | For each variable of interest, give sources of data and details of methods of assessment (measurement). Describe comparability of assessment methods if there is more than one group |  |  |
| Bias | 9 | Describe any efforts to address potential sources of bias | 5, 9 | Immortal time bias – only the years when an individual had a dental check-up were included into incidence analysis. Selection bias – the coverage rate was acceptable, but the caution should be taken when extrapolating the findings. |
| Study size | 10 | Explain how the study size was arrived at | N/A |  |

| Quantitative variables | 11 | Explain how quantitative variables were handled in the analyses. If applicable, describe which groupings were chosen and why | 6 |  |
| --- | --- | --- | --- | --- |
| Statistical methods | 12 | (*a*) Describe all statistical methods, including those used to control for confounding | 6 | Generalized estimating equation models with logit link function were used to analyze the prevalence of jaw catching/locking, with age as an independent variable. Age was modelled using natural cubic splines with five knots. |
|  |  | (*b*) Describe any methods used to examine subgroups and interactions | 5-6 | Descriptive statistics were used to characterize the study population and to estimate the relationship between the onset of jaw catching/locking and/or orofacial pain. |
|  |  | (*c*) Explain how missing data were addressed | 5 | In the sample for the present study, 13 percent of the data were missing and therefore left out from the analysis. |
|  |  | (*d*) *Cohort study*—If applicable, explain how loss to follow-up was addressed  *Case-control study*—If applicable, explain how matching of cases and controls was addressed  *Cross-sectional study*—If applicable, describe analytical methods taking account of sampling strategy | N/A |  |
|  |  | (*e*) Describe any sensitivity analyses | N/A |  |
| Results | | | | |
| Participants | 13* | (a) Report numbers of individuals at each stage of study—eg numbers potentially eligible, examined for eligibility, confirmed eligible, included in the study, completing follow-up, and analysed | 5, 6, Figure 1 | In total, 180,308 individuals (equal gender distribution) were examined in 525,707 dental examinations (median age at examination: 29.0 years, IQR 16-29 years), with a median number of three examinations per individual over the study period. |
|  |  | (b) Give reasons for non-participation at each stage | N/A |  |
|  |  | (c) Consider use of a flow diagram | Figure 1 |  |
| Descriptive data | 14* | (a) Give characteristics of study participants (eg demographic, clinical, social) and information on exposures and potential confounders | 5, Table 1 | In total, 180,308 individuals (equal gender distribution) were examined in 525,707 dental examinations (median age at examination: 29.0 years, IQR 16-29 years), with a median number of three examinations per individual over the study period. |
|  |  | (b) Indicate number of participants with missing data for each variable of interest | N/A |  |
|  |  | (c) *Cohort study*—Summarise follow-up time (eg, average and total amount) | 5 | Because the follow-up was on annual basis for very few individuals, there was a potential risk of immortal time bias. Therefore, only years when each individual had an examination contributed in the calculation of total person-years. In text: An annual follow-up was rare, therefore, only years when an individual had an examination contributed to the calculation of total person-years. |
| Outcome data | 15* | *Cohort study*—Report numbers of outcome events or summary measures over time | Table 2 |  |
|  |  | *Case-control study—*Report numbers in each exposure category, or summary measures of exposure |  |  |
|  |  | *Cross-sectional study—*Report numbers of outcome events or summary measures |  |  |
| Main results | 16 | (*a*) Give unadjusted estimates and, if applicable, confounder-adjusted estimates and their precision (eg, 95% confidence interval). Make clear which confounders were adjusted for and why they were included | Table 1 and 2 | 95% CI |
|  |  | (*b*) Report category boundaries when continuous variables were categorized | N/A |  |
|  |  | (*c*) If relevant, consider translating estimates of relative risk into absolute risk for a meaningful time period |  |  |

| Other analyses | 17 | Report other analyses done—eg analyses of subgroups and interactions, and sensitivity analyses | 5 | Incidence subcohort and onset subcohort. |
| --- | --- | --- | --- | --- |
| Discussion | | | | |
| Key results | 18 | Summarise key results with reference to study objectives | 8 | Exclusive onsets; higher incidence, prevalence, and persistence of jaw catching/locking in women than in men. |
| Limitations | 19 | Discuss limitations of the study, taking into account sources of potential bias or imprecision. Discuss both direction and magnitude of any potential bias | 8,9 |  |
| Interpretation | 20 | Give a cautious overall interpretation of results considering objectives, limitations, multiplicity of analyses, results from similar studies, and other relevant evidence | 9-11 |  |
| Generalisability | 21 | Discuss the generalisability (external validity) of the study results | 8 | In this setting, we found a coverage of more than half of the population in Västerbotten, which is well in line with acceptable coverage for surveys in general of 50-60% (Nulty 2008). The frequency of the routine dental examinations in Sweden varies from approximately once per year to once every two years and depends not only on a patient’s own initiative but also on the dental health of the patient as determined by the dentist. In the present study, the median number per individual was three dental examinations over the eight-year period. Extrapolation of the findings outside the study’s follow-up period should be done with caution, but collectively we believe our results are generalizable for comparable settings. |
| Other information | |  | | |
| Funding | 22 | Give the source of funding and the role of the funders for the present study and, if applicable, for the original study on which the present article is based | 12 | This study was supported by the Swedish Dental Society and through regional agreement between Umeå University and the Region of Västerbotten in cooperation in the fields of Medicine, Odontology and Health. |

*Give information separately for cases and controls in case-control studies and, if applicable, for exposed and unexposed groups in cohort and cross-sectional studies.

**Note:** An Explanation and Elaboration article discusses each checklist item and gives methodological background and published examples of transparent reporting. The STROBE checklist is best used in conjunction with this article (freely available on the Web sites of PLoS Medicine at http://www.plosmedicine.org/, Annals of Internal Medicine at http://www.annals.org/, and Epidemiology at http://www.epidem.com/). Information on the STROBE Initiative is available at www.strobe-statement.org.
